# Age-Specific Seasonal Influenza Vaccine Effectiveness against Different Influenza Subtypes in the Hospitalized Population in Lithuania during the 2015–2019 Influenza Seasons

**DOI:** 10.3390/vaccines9050455

**Published:** 2021-05-04

**Authors:** Monika Kuliese, Aukse Mickiene, Ligita Jancoriene, Birute Zablockiene, Giedre Gefenaite, Study Group

**Affiliations:** 1 Department of Infectious Diseases, Lithuanian University of Health Sciences, Baltijos Street 120, 47116 Kaunas, Lithuania; aukse.mickiene@lsmuni.lt (A.M.); giedre.gefenaite@med.lu.se (G.G.); 2 Clinic of Infectious Diseases and Dermatovenerology, Faculty of Medicine, Institute of Clinical Medicine, Vilnius University, Santariskiu Street 14, 08406 Vilnius, Lithuania; ligita.jancoriene@santa.lt (L.J.); birute.zablockiene@santa.lt (B.Z.); 3 Center of Infectious Diseases, Vilnius University Hospital Santaros Klinikos, Santariskiu Street 14, 08406 Vilnius, Lithuania; 4 Department of Health Sciences, Faculty of Medicine, Lund University, Box 157, 22100 Lund, Sweden

**Keywords:** hospital surveillance, risk groups, older people, underlying conditions, influenza, severe outcomes, laboratory-confirmed

## Abstract

Background: Continuous monitoring of seasonal influenza vaccine effectiveness (SIVE) is needed due to the changing nature of influenza viruses and it supports the decision on the annual update of vaccine composition. Age-specific SIVE was evaluated against different influenza subtypes in the hospitalized population in Lithuania during four influenza seasons. Methods: A test-negative case-control study design was used. SIVE and its 95% confidence intervals (95% CI) were calculated as (1 – odds ratio (OR)) × 100%. Results: Adjusted SIVE in 18–64-year-old individuals against influenza A, A(H1N1)pdm09 and B/Yamagata were 78.0% (95% CI: 1.7; 95.1%), 88.6% (95% CI: −47.4; 99.1%), and 76.8% (95% CI: −109.9; 97.4%), respectively. Adjusted SIVE in individuals aged 65 years and older against influenza A, influenza B, and B/Yamagata were 22.6% (95% CI: −36.5; 56.1%), 75.3% (95% CI: 12.2; 93.1%) and 73.1% (95% CI: 3.2; 92.5%), respectively. Unadjusted SIVE against influenza A(H3N2) among 18–64-year-old patients was 44.8% (95% CI: −171.0; 88.8%) and among those aged 65 years and older was 5.0% (95% CI: −74.5; 48.3%). Conclusions: Point estimates suggest high SIVE against influenza A in 18–64-year-old participants, and against influenza B and B/Yamagata in those 65 years old and older.

## 1. Introduction

According to the World Health Organization (WHO), the number of patients with severe acute respiratory infection (SARI), mainly caused by influenza viruses, increases during autumn and winter seasons in the Northern Hemisphere [1]. The highest mortality rates due to influenza and its complications are registered among older adults [2], possibly because of immunosenescence that occurs during the process of ageing and results in less effective and slower immune response and underlying medical conditions [3]. Despite its limitations, the most effective specific measure to prevent severe influenza disease is annual vaccination [4].

The National Program of Immunoprophylaxis in Lithuania recommends that all healthcare workers, pregnant women, institutionalized people, persons with underlying medical conditions, and aged over 65 years receive annual influenza vaccination [5]. This is covered by the state. While the WHO suggests vaccinating at least 75% of older persons against seasonal influenza due to their high risk of developing severe influenza [6], their vaccination uptake in Lithuania remains one of the lowest in Europe [7]. However, based on the absolute number of vaccinated individuals of 65 years and older reported by the Center for Communicable Diseases and AIDS and the corresponding population of this age group based on Statistics Lithuania, influenza vaccination uptake in older people over the last decade increased from 9% during the influenza season 2010–2011 to 19% in 2019–2020.

Due to the changing nature of influenza viruses and the need for the annual update of the influenza vaccine composition, continuous monitoring of its effectiveness in different settings is necessary to inform public health. Furthermore, diversity in the timing of influenza seasons, as well as influenza spread patterns in the WHO European Region [8] further advocates geographically distributed SIVE assessments to strengthen public health planning and action related to influenza preparedness on the national as well as the regional level. We therefore assessed the seasonal influenza vaccine effectiveness between 2015 and 2019 in a hospital setting in Lithuania among several risk groups recommended for annual vaccination.

## 2. Materials and Methods

### 2.1. Objectives

The primary objective of this study was to measure age-specific SIVE against laboratory-confirmed influenza hospitalization during four influenza seasons in Lithuania. The secondary objectives were to estimate SIVE by influenza type and subtype.

### 2.2. Study Design, Population, Setting, and Recruitment Procedure

This was a hospital based test-negative case-control (TND) study conducted in Lithuania during four influenza seasons (2015–2016, 2016–2017, 2017–2018, and 2018–2019).

The study population was community-dwelling individuals aged 18 years and older eligible for influenza vaccination according to the national legislation [5]. Influenza vaccination is, among others, recommended and provided by the state to pregnant women, healthy 65 years and older individuals, and those 18 years and older with at least one underlying medical condition (e.g., cancer, lung, heart, rheumatologic, kidney, endocrine diseases or diabetes, immunodeficiency and transplantation, anemia, dementia, or stroke; for the International Classification of Diseases 10 codes, see Appendix A Table A1).

Patients were recruited into the study if they were hospitalized to the Departments of Infectious Diseases, Geriatrics, or Internal Medicine in Kaunas Hospital of Lithuanian University of Health Sciences or the Center of Infectious Diseases in Vilnius University Hospital Santaros Klinikos. The catchment population of these two hospitals serves approximately 13% (312,557) of the 18-years-and-older population [9,10]. The infectious diseases units in these hospitals are the main centers where patients with clinically suspected influenza are admitted in Vilnius and Kaunas counties.

A patient with SARI was characterized as an individual hospitalized for at least 24 h with at least one systemic symptom (fever, myalgia, malaise, headache, or general deterioration) and at least one respiratory symptom (cough, sore throat, or shortness of breath). The inclusion criteria were eligibility for influenza vaccination, a swab taken less than seven days after the self-reported SARI onset and within 48 h after admission to hospital, no positive influenza test in the current influenza season, willingness to participate, and ability to communicate. Institutionalized participants were excluded due to different outcome and exposure risks. After collecting demographic and clinical information from self-reports and medical documents, a study clinician took one nasopharyngeal and one throat specimen for multiplex reverse transcription polymerase chain reaction (RT-PCR) testing.

### 2.3. Outcome

The outcome of interest was laboratory-confirmed influenza illness in persons hospitalized with SARI. Persons with SARI who were positive for any type of laboratory-confirmed influenza were defined as influenza cases. Patients with SARI who were negative for any influenza virus were influenza-negative controls.

### 2.4. Exposure

The exposure of interest was vaccination with seasonal influenza vaccine during 2015–2016, 2016–2017, 2017–2018, and 2018–2019 influenza seasons. Participants were considered as vaccinated if the vaccine was administered more than 14 days before the SARI symptoms onset and vaccination status was verified with the general practitioner (GP) or vaccination pass. Vaccine composition and vaccines available free-of-charge for the risk group patients in Lithuania in each season are provided in Table 1.

### 2.5. Covariates

Information about age, sex, length of hospitalization, antiviral drug use, and admission to the critical care unit were collected from the medical documents. Smoking status, weight, height, presence of underlying health conditions, number of hospital admissions in the latest year due to the exacerbation of the underlying conditions (but not repeated prescriptions), socioeconomic status (highest education, employment), and living area (urban/rural) were collected through the self-reports. Body mass index (BMI) was calculated as kg/m^2^ and obesity was defined as BMI ≥ 30. Pre-hospitalization Barthel index to measure performance in activities of daily living (ADL) [11] was collected by a combination of an interview and observation.

### 2.6. Laboratory Analysis

Nasopharyngeal and throat swabs were tested with multiplex RT-PCR for influenza and other respiratory viruses with Aniplex IIRV16 Detection (V1.1) (Seegene) kit. Influenza-positive samples were subtyped to influenza A(H1N1)pdm09, influenza A(H3N2), influenza B/Yamagata, or influenza B/Victoria. The laboratory analysis, in more detail, is described elsewhere [12].

### 2.7. Statistical Analysis

For descriptive analysis Fisher’s exact test, Pearson’s Chi-square test, Student’s *t*-test, and Mann–Whitney test were performed. SIVE and its 95% confidence interval (95% CI) was estimated by using the formula (1 − odds ratio) × 100%. The odds ratio in this analysis is the ratio between the odds that an outcome will occur among the vaccinated, compared to the odds of the outcome occurring among the unvaccinated. The analysis was adjusted for a set of confounders if, after adjustment for each of the potential confounders separately, it changed SIVE estimate by ≥10%.

### 2.8. Ethical Considerations

This study was conducted in line with the Lithuanian legislation on research with humans and the Declaration of Helsinki [13,14]. Kaunas Regional Biomedical Research Ethics Committee approvals were obtained for each season (No. P2-158200-04-476-138/2012, No. P3-158200-04-476-138/2012, No. P4-158200-04-476-138/2012, No. P5-158200-04-476-138/2012, P6-158200-04-476-138/2012). All study participants gave written informed consents (IC).

### 2.9. Sample Size Calculation

To achieve the statistical power of 80% with a confidence level of 95%, with the vaccination rates of 3.3% and 11.1% among 18–64-year-old cases and controls, respectively, the required sample size for unadjusted analysis should be at least 70 participants. In the older group, due to higher vaccination rates (10.2% vs. 14.7% in cases and controls, respectively), the sample size required should be at least 28 participants. The sample size calculations were performed with OpenEpi, Version 3.

### 2.10. Sample

**Figure 1:**
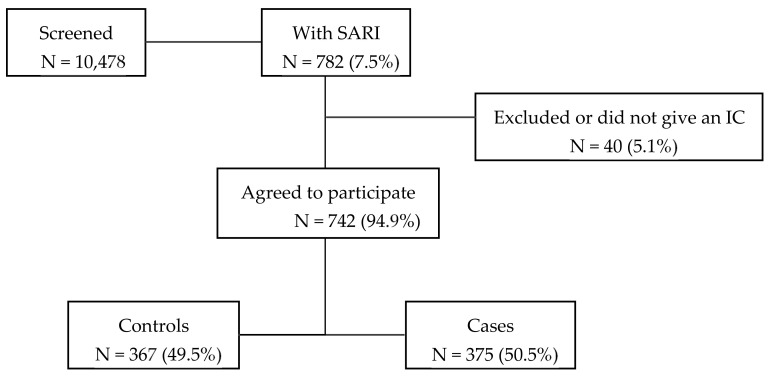
Flowchart of the recruitment of patients.

During 2015–2019 influenza seasons, 10,478 patients hospitalized in one of the participating departments were screened for inclusion (Figure 1). Of these, 782 (7.5%) of the patients were identified as having SARI symptoms, of which 94.9% met the inclusion criteria, gave an informed consent, and were recruited into the study.

## 3. Results

Of the 742 included participants, 375 (50.5%) of the subjects tested positive for influenza and 367 (49.5%) were influenza-negative controls. (Figure 1). Throughout the entire study influenza A was predominant (34.4%) with influenza A(H3N2) being identified the most often. The second most common influenza subtype detected was influenza B/Yamagata (Table 2).

During the influenza season 2015–2016 influenza A(H1N1pdm09) was predominant, while influenza A(H3N2) dominated during the 2016–2017 and 2018–2019 influenza seasons (Table 2). Influenza B/Yamagata circulated most often during the 2017–2018 season.

Among 18–64-year-old influenza cases, there were fewer underlying medical conditions such as dementia, stroke, and chronic cardiovascular diseases (Table 3). Compared to controls, cases were more often prescribed antivirals, but were less obese. SIV coverage among the cases was statistically significantly lower than among the controls in the current (3.3% vs. 11.1%), as well as the previous (1.7% vs. 10.1%) season. Pregnancy was more common among cases.

Among the participants of 65 years and older, influenza cases had less nutritional deficiency and underlying cardiovascular and lung diseases than controls (Table 3). Influenza cases were less often hospitalized due to the exacerbations of the underlying medical conditions during the previous year. Furthermore, cases were prescribed antivirals more often as compared to controls and on average, were hospitalized two days less. Approximately one third of the study sample received antivirals on the day of swabbing, with about 50% being swabbed one day after starting with antivirals.

Out of 81 detected other respiratory viruses (parainfluenza, adenovirus, rhinovirus, metapneumovirus, bocavirus, coronavirus, respiratory syncytial virus, and enterovirus), 11 were influenza coinfections, of which four were influenza A(H1N1)pdm09, two influenza B/Yamagata, three influenza A (untyped), one influenza A(H3N2), and one in B/Victoria cases. In both age groups, cases were diagnosed with other viral respiratory infections less frequently than controls.

In the total sample, 15/742 (2%) participants died during the hospitalization. Four deaths occurred among influenza cases in the 18–64-year-old individuals, one of which was vaccinated. Out of 11 deaths in people 65 years and older, four were unvaccinated confirmed-influenza cases and among seven controls, two were vaccinated.

### Vaccine Effectiveness Analysis

Adjusted SIVE against any influenza in 18–64 years old group was 71.9% (Table 4), while adjusted SIVE point estimate among those 65 years old and older was 26.9%. In 18–64-year-old participants the adjusted SIVE against influenza A was 78.0%, while among 65 years old and older persons it was 22.6%. After adjustment, the SIVE point estimate against influenza A(H1N1)pdm09 in 18–64-year-old participants slightly increased, but it did not reach statistical significance. It was not possible to estimate the adjusted SIVE against influenza A(H1N1)pdm09 in people of 65 years and older, or against influenza A(H3N2) (Table 4).

SIVE estimates among people aged 18–64 slightly decreased after adjustment against influenza B (78.4% vs. 74.1%) and slightly increased against influenza B/Yamagata (73.3% vs. 76.8%), but were not statistically significant. In people of 65 years and older SIVE estimates against influenza B and B/Yamagata after adjustment decreased slightly (77.9% vs. 75.3% and 74.4% vs. 73.1%, respectively) and remained statistically significant.

## 4. Discussion

This study was conducted to measure age-specific SIVE against different laboratory-confirmed influenza types and subtypes during four influenza seasons (2015–2019). SIVE point estimates against any influenza were higher among persons aged 18–64 years (72%) compared to people aged 65 and above (27%), however the latter did not reach statistical significance. Similar results were observed against different influenza A subtypes. The highest statistically significant adjusted SIVE was found against influenza B and B/Yamagata among persons aged 65 and above (75% and 73%, respectively); similar but not statistically significant SIVE was found in 18–64-year-old individuals.

According to the WHO European Region virological influenza surveillance data, influenza A(H1N1)pdm09 dominated during 2015–2016 and 2018–2019 influenza seasons [15,16], which was also found in our study. Antigenic and genetic data showed that regardless of the evolving influenza A(H1N1)pdm09 viruses from their 2009 ancestor, during both seasons most of the circulating influenza A(H1N1)pdm09 viruses were similar to the viruses included in the seasonal vaccines in the respective seasons. Circulated and vaccine strain match might explain higher than moderate SIVE point estimates against influenza A(H1N1)pdm09 among persons 18–64 years old in our study. However, in 65-year-old and older participants, lower and not statistically significant adjusted and unadjusted SIVE against influenza A and A(H1N1)pdm09 was found. Similar SIVE estimates were found among the hospitalized adults in the study conducted in the US [17]. For example, SIVE against any laboratory-confirmed influenza and influenza A(H1N1)pdm09 in people of 18–49 years old was found to be 77% and 67%, and in the age group of 65 years and older, 51% and 63%, respectively. In addition, lower SIVE estimates against any influenza and A(H1N1)pdm09 were reported among hospitalized older people as compared with patients aged 18–64 years across Europe [18,19,20]. For instance, SIVE against any influenza and influenza A(H1N1)pdm09 reported by the European Development of Robust and Innovative Vaccine Effectiveness (DRIVE) network among patients aged 18–64 were 40% and 51%, and among persons 65 years old and older, 27% and 35%, respectively [18]. Similarly, SIVE against any influenza and influenza A(H1N1)pdm09 in Northern Spain among hospitalized older adults was 26% and 47% [19]. In addition, 20% SIVE was reported by the Valencia Hospital Network against influenza A(H1N1)pdm09 hospitalizations in patients 60 years old or older [20]. Possibly, lower SIVE against influenza A(H1N1)pdm09 in older age groups could be explained by lower incidence of influenza A(H1N1)pdm09 due to previous exposure [21], immunosenescence, or, given rather low absolute numbers of cases, we also cannot rule out residual confounding.

In our study, influenza A(H3N2) was the predominant strain during the influenza seasons 2016–2017 and 2018–2019, which is also in line with the European Centre for Disease Prevention and Control (ECDC) and WHO European region reports [16,22,23]. Our SIVE estimates against influenza A(H3N2) are in line with other studies in hospitalized populations. Low SIVE point estimates against influenza A(H3N2) were reported by the DRIVE as 2% among 18–64-year-old patients and 12% among persons 65 years old and older [18]. Consistent SIVE estimates were found in Denmark against influenza A(H3N2) among hospitalized patients aged 65 years old and older (7%) [24], as well as in other studies across Europe among hospitalized population [19,25,26,27]. Genetic characterization of the early 2016–2017 season viral isolates from 24 European countries revealed that the highest proportion of the influenza A(H3N2) viruses belonged to the 3C.2a clade, represented by A/Hong Kong/4801/2014 [23]. Analysis showed that the greater part of the detected 3C.2a viruses belonged to a new manifesting subclade 3C.2a1, represented by A/Bolzano/7/2016. Despite these findings, circulating viruses within the 3C.2a1 subclade were assumed as antigenically similar to the seasonal Northern Hemisphere vaccine component, A/Hong Kong/4801/2014. However, around 30% of analyzed A(H3N2) viruses could not be assigned to an antigenic reporting category, which points out technical difficulties or antigenic substitution of the circulating strains and might be related to lower SIVE estimates. Moreover, during the influenza season 2018–2019, several genetic subclades of influenza A(H3N2) viruses circulated with confirmed genetic diversity [16] of which clade 3C.3a accounted for one-fifth of viruses and was considered as being antigenically distinct from the Northern Hemisphere vaccine strain A/Singapore/INFIMH-16–0019/2016 (H3N2)-like virus. Low influenza A(H3N2) SIVE estimates in our study could probably be explained by reported genetic and antigenic mismatch between circulating and the vaccine A(H3N2) strains. Another possible impact of lower SIVE against influenza A(H3N2) might be related with the egg-adaptive mutations that the virus obtained during the production of candidate vaccine virus, which affected its antigenicity [28]. In addition, existing evidence suggests that previous seasonal influenza vaccination may influence current season SIVE and lead to lower protection against influenza A(H3N2) during the current season and antigenic distance hypothesis cannot be excluded [29,30]. Due to low sample size, we were not able to run SIVE analysis adjusted to previous vaccinations, and therefore could not test this hypothesis in the current study.

Our study results suggest protection against influenza B and B/Yamagata, in 65 years and older and 18–64-year-old participants (the former not statistically significant). Similar SIVE against influenza B has been reported in the out- and inpatient settings in Spain (67%) [31] and Hong Kong SAR (77% and 71% in 18–64 and 65 years and older groups, respectively) [32]. On the contrary, low end-of-season protection against influenza B and B/Yamagata were reported in the US and the UK in the primary care setting [33,34]. As reported by the ECDC and the WHO, influenza B was dominant in Europe during the 2017–2018 influenza season [35,36], which was also reflected by our study where 80% of influenza cases were confirmed with B/Yamagata. In the Northern Hemisphere, however, the 2017–2018 trivalent SIV contained influenza B/Brisbane/60/2008-like (B/Victoria lineage) antigens, which points to the mismatch between the circulating and vaccine strain virus. Even though influenza B/Victoria and B/Yamagata lineages are antigenically diverse, it is reported that these strains are more genetically similar than influenza A(H1N1)pdm09 and influenza A(H3N2) [37]. It was found that influenza B/Victoria and B/Yamagata shared more than 90% amino acid affinity within hemagglutinin and neuraminidase proteins than influenza A subtypes with less than 45% similarity of these proteins [37]. Along with previous reports [38,39], high SIVE found in our study also suggests possible cross-lineage protection between different influenza B strains from the past seasons.

Some suggest that reduced SIVE estimates in older population might be explained by the effect of immunosenescence and weakened immune system [3,40,41]. Due to different immunity characteristics across different ages and older people being more at risk for severe influenza [3,40], we performed stratified-by-age SIVE analysis with different sets of confounders for each group. The estimates against influenza A and its subtypes across the age groups differed substantially, with two- to nine-fold lower SIVE point estimates among the older people. To better protect older adults against influenza, the results of our study would be supportive of vaccination with higher antigenic doses and adjuvanted vaccines [42]. On the other hand, SIVE against influenza B estimates in our study suggested similar and good protection in both age groups, which would suggest that despite age-related immune system changes seasonal influenza vaccination remains effective and has protective benefit in older adults as well [43,44].

### Limitations and Strengths

Several limitations and strengths of our study must also be addressed. First, the low number of vaccinated individuals led to broad confidence intervals of some of the SIVE estimates and lack of power to run adjusted analysis against influenza A(H3N2) in both age groups and against influenza A(H1N1)pdm09 in those aged 65 years and above. Still, the estimates in our study were quite consistent before and after adjustment, and in line with other SIVE studies across and beyond Europe. Secondly, due to lack of electronic access to patient’s outpatient records (e.g., population-based health register), information about the underlying medical conditions and their severity was collected through self-reports. While it is likely that chronic diseases would be reported correctly and in some cases could be validated with the medical records, the severity (e.g., number of hospital visits in the preceding 12 months) is a lot harder to recall, which may lead to bias. Most of the health-related information relating to the current hospitalization, including prescriptions, however, came from the hospital records and is therefore accurate. When looking into effect changes to select potential confounders, we identified and therefore controlled for different sets of potential confounders for different influenza subtypes and age groups. More detailed analyses on the effects on SIVE of underlying medical conditions, however, was not possible due to lack of statistical power. Next, influenza infection was confirmed with highly specific molecular assay, which reduced the probability of false-negative results. The participants were recruited into the study within seven days after the onset of SARI symptoms, when the viral shedding was the highest [45]. Additionally, 474 (63.9%) of our study subjects were prescribed antivirals, yet the vast majority of them were swabbed within one day after antiviral administration, which also reduce the chance of false negative results [46]. Selection bias was minimized by the recruitment procedure, as SARI cases were recruited into the study before the swab was taken and laboratory results were available to the patient and the researcher. Vaccination status was validated with the patient’s GP. While unmeasured confounding cannot be excluded, we are confident that the overall exposure, outcome, and covariate information in our study has low risk of bias. Last, but not least, test-negative case-control design to measure SIVE is a widely used method with well-explored strengths, such as being less vulnerable to bias due to false classification of infection and the confounding by healthcare seeking behavior [47,48,49].

## 5. Conclusions

The point estimates of >70% found in this study suggest high SIVE against any influenza in 18–64-year-old participants, and against influenza B and B/Yamagata in those 65 years old and older. Despite high SIVE against influenza B even the circulating subtypes did not match the viruses included in the vaccine during the seasons 2015–2017 and 2018–2019, the results of our study suggest that the use of the quadrivalent vaccines including two subtypes of influenza B may be beneficial.

## Figures and Tables

**Table 1:** Free-of-charge vaccines used to vaccinate risk group patients in Lithuania in the 2015–2019 influenza seasons.

Influenza Season	Vaccine Composition	Vaccine Type
2015–2016	- California/7/2009(H1N1)-like - A/Switzerland/9715293/2013(H3N2)-like - B/Phuket/3073/2013-like (B/Yamagata lineage)	Trivalent inactivated influenza vaccine, non-adjuvanted
2016–2017	- California/7/2009(H1N1)-like - A/Hong Kong/4801/2014(H3N2)-like - B/Brisbane/60/2008-like (B/Victoria lineage)	Trivalent inactivated influenza vaccine, non-adjuvanted
2017–2018	- A/Michigan/45/2015 (H1N1)pdm09-like - A/Hong Kong/4801/2014 (H3N2)-like - B/Brisbane/60/2008-like (B/Victoria lineage)	Trivalent inactivated influenza vaccine, non-adjuvanted
2018–2019	- A/Michigan/45/2015 (H1N1)pdm09-like - A/Singapore/INFIMH-16-0019/2016 (H3N2)-like - B/Colorado/06/2017-like (B/Victoria/2/87-lineage)	Trivalent inactivated influenza vaccine, non-adjuvanted

**Table 2:** Influenza types and subtypes in the total sample during the 2015–2019 influenza seasons.

Influenza Season	Controls	A(H1N1pdm09)	A(H3N2)	A (Untyped)	B/Yamagata	B/Victoria	B (Untyped)
	N (%)	N (%)	N (%)	N (%)	N (%)	N (%)	N(%)
Season 2015–2016	91 (55.8)	50 (30.7)	–	14 (8.6)	–	7 (4.3)	1 (0.6)
Season 2016–2017	104 (53.9)	–	83 (54.6)	–	5 (2.6)	–	1 (0.5)
Season 2017–2018	91 (42.9)	5 (2.4)	8 (3.8)	2 (0.9)	97 (45.8)	2 (0.9)	7 (3.3)
Season 2018–2019	81 (46.6)	29 (16.7)	61 (35.1)	3 (1.7)	–	–	–
All seasons	367 (49.5)	84 (11.3)	152 (20.5)	19 (2.6)	102 (13.7)	9 (1.2)	9 (1.2)

**Table 3:** Demographic and clinical features of influenza cases and controls and by age-group in the total sample during the 2015–2019 influenza seasons.

Variables	Age Group 18–64		*p*-Value	Age Group ≥65		*p*-Value
	Influenza-Positive N = 121 (40.1%)	Influenza-Negative N = 81 (59.9%)		Influenza-Positive N = 254 (47.0%)	Influenza-Negative N = 286 (53.0%)	
**Influenza season**						
Season 2015–2016	40 (33.1)	31 (38.3)	0.057 ^a^	32 (12.6)	60 (21.0)	0.052 ^a^
Season 2016–2017	17 (14.0)	21 (25.9)		72 (28.3)	83 (29.0)	
Season 2017–2018	38 (31.4)	15 (18.5)		83 (32.7)	76 (26.6)	
Season 2018–2019	26 (21.5)	14 (17.3)		67 (26.4)	67 (23.4)	
Male	61 (50.4)	40 (49.4)	0.886 ^a^	108 (42.5)	131 (45.8)	0.443 ^a^
Urban	84 (69.4)	51 (75.3)	0.362 ^a^	195 (76.8)	237 (82.9)	0.077 ^a^
Age (median, min; max)	54.0 (19; 64)	54.0 (21; 64)	0.692 ^d^	77.0 (65; 96)	76.0 (65; 92)	0.274 ^d^
Education						
High (college, university)	57 (47.9)	34 (42.5)	0.453 ^a^	59 (23.4)	64 (22.4)	0.775 ^a^
Low (primary, unfinished, secondary, professional)	62 (52.1)	46 (57.5)		193 (76.6)	222 (77.6)	
Occupation						
Intellectual or/and physical work	62 (51.2)	46 (56.8)	0.438 ^a^	18 (7.1)	15 (5.2)	0.372 ^a^
Retired/handicapped/jobless	59 (48.8)	35 (43.2)		236 (92.9)	271 (94.8)	
Smoking						
Never	50 (41.3)	35 (43.2)	0.512 ^a^	172 (68.0)	185 (64.7)	0.447 ^a^
Former	31 (25.6)	25 (30.9)		60 (23.7)	81 (28.3)	
Current	40 (33.1)	21 (25.9)		21 (8.3)	20 (7.0)	
Pregnant	17 (14.0)	4 (4.9)	0.038 ^a^	–	–	–
Chronic condition (at least 1)	109 (90.1)	77 (95.1)	0.199 ^a^	221 (87.0)	269 (94.1)	0.005 ^a^
At least one hospitalization due to exacerbation of underlying conditions in the previous 12 months	22 (18.3)	22 (27.2)	0.138 ^a^	62 (24.6)	123 (43.0)	<0.001 ^a^
Cardiovascular diseases	37 (30.6)	37 (45.7)	0.029 ^a^	167 (65.7)	223 (78.0)	0.002 ^a^
Lung diseases	29 (24.0)	25 (30.9)	0.278 ^a^	52 (20.5)	87 (30.4)	0.008 ^a^
Endocrine diseases, diabetes	23 (19.0)	17 (21.0)	0.729 ^a^	46 (18.1)	58 (20.3)	0.523 ^a^
Renal diseases	6 (5.0)	2 (2.5)	0.480 ^b^	27 (10.6)	43 (15.0)	0.128 ^a^
Immunodeficiency and transplantations	12 (9.9)	6 (7.4)	0.539 ^a^	5 (2.0)	2 (0.7)	0.262 ^b^
Rheumatologic diseases	6 (5.0)	2 (2.5)	0.480 ^b^	8 (3.1)	9 (3.1)	0.999 ^a^
Dementia, stroke	4 (3.3)	9 (11.1)	0.027 ^a^	63 (24.8)	81 (28.3)	0.356 ^a^
Hematologic cancer	7 (5.8)	1 (1.2)	0.148 ^b^	7 (2.8)	9 (3.1)	0.789 ^a^
Non-hematologic cancer	14 (11.6)	7 (8.6)	0.504 ^a^	36 (14.2)	39 (13.6)	0.857 ^a^
Anemia, spleen pathology	12 (9.9)	6 (7.4)	0.539 ^a^	15 (5.9)	29 (10.1)	0.073 ^a^
Cirrhosis	2 (1.7)	4 (4.9)	0.221 ^b^	5 (2.0)	2 (0.7)	0.262 ^b^
Nutritional deficiency	4 (3.3)	0 (0.0)	0.151 ^b^	6 (2.4)	17 (5.9)	0.040 ^a^
Obesity	38 (31.4)	37 (45.7)	0.040 ^a^	80 (31.5)	87 (30.4)	0.787 ^a^
BMI (median, min; max)	26.3 (17.3; 51.7)	29.2 (18.7; 45.2)	0.020 ^d^	27.4 (17.3; 43.0)	27.8 (15.9; 50.0)	0.468 ^d^
Any antiviral use	114 (94.2)	48 (59.3)	<0.001 ^a^	204 (80.3)	108 (37.8)	<0.001 ^a^
Statin use	8 (12.5)	2 (6.9)	0.419 ^a^	18 (12.0)	13 (9.0)	0.418 ^a^
Other respiratory virus detected	5 (4.1)	14 (17.7)	0.001 ^a^	6 (2.4)	56 (19.6)	<0.001 ^a^
SIV in current season	4 (3.3)	9 (11.1)	0.027 ^a^	26 (10.2)	42 (14.7)	0.120 ^a^
SIV in previous season	2 (1.7)	8 (10.1)	0.008 ^a^	20 (7.9)	30 (10.5)	0.309 ^a^
Transfer to the intensive-care unit	10 (8.3)	2 (2.5)	0.088 ^a^	13 (5.1)	10 (3.5)	0.352 ^a^
Length of hospitalization (mean, SD)	6.5 (5.0)	6.7 (3.2)	0.721 ^c^	7.7 (3.9)	9.8 (5.1)	<0.001 ^c^
Barthel score before the SARI hospitalization (mean, SD)	99.8 (1.6)	96.3 (15.6)	0.051 ^c^	94.3 (17.1)	92.7 (16.4)	0.267 ^c^
Days between the SARI onset and swab (median, min; max)	4.0 (0; 7)	4.0 (0; 7)	0.910 ^d^	4.0 (0; 7)	4.0 (0; 7)	0.126 ^d^
Days between antivirals use and swab						
0 days	36 (31.6)	13 (27.1)	0.191 ^a^	64 (31.4)	26 (24.1)	0.180 ^a^
1 day	61 (53.5)	22 (45.8)		111 (54.4)	59 (54.6)	
2 and more days	17 (14.9)	13 (27.1)		29 (14.2)	23 (21.3)	
Deaths	4 (3.3)	0 (0.0)	0.151 ^b^	4 (1.6)	7 (2.4)	0.474 ^a^

^a^ Pearson’s Chi-square test; ^b^ Fisher’s exact test; ^c^ Student *t*-test; ^d^ Mann–Whitney test.

**Table 4:** Seasonal influenza vaccine effectiveness against different laboratory-confirmed influenza subtypes by age groups during the 2015–2019 influenza seasons.

	Influenza-Positive	Influenza-Negative	Unadjusted SIVE% (95% CI)	*p*-Value of the Unadjusted Model	Adjusted SIVE% (95% CI)	*p*-Value of the Adjusted Model	R Square of the Adjusted Model
	Vaccinated/Total (%)	Vaccinated/Total (%)					
Any influenza							
Age 18–64 years	4/121 (3.3)	9/81 (11.1)	72.7 (7.9 to 91.9)	0.036	71.9 ^a^ (−1.9 to 92.2)	0.053	0.090
Age ≥ 65 years	26/254 (10.2)	42/286 (14.7)	33.8 (−11.6 to 60.7)	0.122	26.9 ^b^ (−24.4 to 57.0)	0.249	0.043
Influenza A							
Age 18–64 years	3/83 (3.6)	9/81 (11.1)	70.0 (−15.1 to 92.2)	0.079	78.0 ^c^ (1.7 to 95.1)	0.047	0.150
Age ≥ 65 years	23/173 (13.3)	42/286 (14.7)	10.9 (−54.0 to 48.5)	0.679	22.6 ^d^ (−36.5 to 56.1)	0.376	0.127
Influenza A(H1N1)pdm09							
Age 18–64 years	1/51 (2.0)	9/81 (11.1)	84.0 (−30.3 to 98.0)	0.087	88.6 ^e^ (−47.4 to 99.1)	0.096	0.141
Age ≥ 65 years	3/34 (8.8)	42/286 (14.7)	43.8 (−92.2 to 83.6)	0.359	N/A	N/A	N/A
Influenza A(H3N2)							
Age 18–64 years	2/31 (6.5)	9/81 (11.1)	44.8 (−171.0 to 88.8)	0.464	N/A	N/A	N/A
Age ≥ 65 years	17/121 (14.0)	42/286 (14.7)	5.0 (−74.5 to 48.3)	0.868	N/A	N/A	N/A
Influenza B							
Age 18–64 years	1/38 (2.6)	9/81 (11.1)	78.4 (−77.2 to 97.4)	0.216	74.1 ^f^ (−146.8 to 97.3)	0.240	0.250
Age ≥ 65 years	3/82 (3.7)	42/286 (14.7)	77.9 (26.9 to 93.4)	0.013	75.3 ^g^ (12.2 to 93.1)	0.030	0.116
Influenza B/Yamagata							
Age 18–64 years	1/31 (3.2)	9/81 (11.1)	73.3 (−119.8 to 96.8)	0.219	76.8 ^h^ (−109.9 to 97.4)	0.194	0.183
Age ≥ 65 years	3/71 (4.2)	42/286 (14.7)	74.4 (14.8 to 92.3)	0.026	73.1 ^i^ (3.2 to 92.5)	0.044	0.093

OR: odds ratio; 95% CI: 95% confidence interval. Adjusted SIVE analysis was adjusted for ^a^ hematologic cancer and Barthel score before the SARI hospitalization; ^b^ hospitalizations due to exacerbation of chronic conditions during previous 12 months; ^c^ influenza season, hematologic cancer, and Barthel score before the SARI hospitalization; ^d^ influenza season; ^e^ pregnancy, hematologic cancer, dementia*/*strokes, anemia, and Barthel score before the SARI hospitalization; ^f^ pregnancy, hematologic cancer, immunodeficiency and transplantations, dementia*/*stroke, anemia, BMI, and hospitalizations due to exacerbation of chronic conditions during previous 12 months; ^g^ cirrhosis, cardiovascular disease, and hospitalizations due to exacerbation of chronic conditions during previous 12 months; ^h^ immunodeficiency and transplantations, renal disease, anemia, BMI, and hospitalizations due to exacerbation of chronic conditions during previous 12 months; ^i^ cirrhosis, cardiovascular disease, and hospitalizations due to exacerbation of chronic conditions during previous 12 months. NA: not applicable.

## Data Availability

The data presented in this study are available in insert article.

## Appendix A

**Table A1:** Underlying medical conditions according to International Classification of Diseases, 10th Edition.

Chronic Underlying Conditions	ICD-10 Codes
Anemia, Spleen pathology	D50–75 S36.0 Q89.01,09
Cancer	C00–96 C7A, B
Cardiovascular conditions	A52.01
	G46
	I00–I09 I20 I21–I23 I24.0,1,8,9 I25 I27–28 134–37 124.1–9 143–6 148 I49 I50 I51–2 O90.3 Q20–28
Cirrhosis	K74
Dementia, stroke	F01–06 F10 F19 G30.9 G31.01,1,09 G91.0–1 G35 G36.0 G37.0–3,5,8,9; G93.0-2,4,5,41,49 I60–64 I67.83 I69
Endocrine disorders, diabetes	E10 E11–14 E15 E16.0–3 E16.8–9 E20–32 E35 E70–79 E83–5,7,8 E89.1
Immunocompromised conditions	D80.0–4,8,9 K71–77, excl. K74 E06.3–5,9 E07 E90 M11 M83 Z94.0–2,5–7 Z95.3 B20–24 R75 Z21
Lung diseases	A15.0 C30.0 C31–2 C33–4 C38.1-4 C39 C45.0,7 D14.0–4 D19.0 I26 J33 J34.1–3,8 J38–9 J43–4 J45–6 J47 J60–70 J80–2,4 J90–1 J92–3,6,8,9 J94 M40–1 M43.8–9 Q30–34 Q65–79 R09.1 Z90.2
Nutritional deficiency	E32.0,1,8,9 E24.0,2,3,8,9 E25.0,8,9 E26.01,102,09,9,81 E34.0 E40,41,43–6 E50,51
Obesity	E65–68
Renal diseases	I12.0,9 M10.30 N00.3,8–9 N01.3 N02.2 N03.2–3,5,8 N04.0,3–4 N05 N07–8 N10–11 N13.30,721,729 N14 N15.0,8 N16 N17–19 N25 N27 N28.9 Q60–4
Rheumatologic diseases	M05.00,10 M06.4,9 M08.00,40 M15.0–2,9 M12,00 M30–31 M32–35

## References

[1] Flu News Europe (2019). Joint ECDC—WHO/Europe Weekly Influenza Update. Summary Weeks 33–39/2019 (12 August–29 September 2019) 2018–2019 Season Overview.

[2] World Health Organization Influenza (Seasonal). https://www.who.int/news-room/fact-sheets/detail/influenza-(seasonal).

[3] Sambhara S., McElhaney J.E. (2009). Immunosenescence and Influenza Vaccine Efficacy. Curr. Top. Microbiol. Immunol..

[4] World Health Organization Influenza vaccination. http://www.euro.who.int/en/health-topics/communicable-diseases/influenza/vaccination.

[5] Lietuvos Respublikos Sveiktos Apsaugos Ministras Įsakymas Dėl Nacionalinės Imunoprofilaktikos 2019–2023 Metų Programos Patvirtinimo. https://e-seimas.lrs.lt/portal/legalAct/lt/TAD/a88940c123b911e9b246d9cc49389932.

[6] World Health Organization The Fifty-sixth World Health Assembly. Prevention and Control of Influenza Pandemics and Annual Epidemics. https://apps.who.int/iris/handle/10665/259836.

[7] European Centre for Disease Prevention and Control (ECDC) (2018). Seasonal Influenza Vaccination and Antiviral Use in EU/EEA Member States. Overview of Vaccine Recommendations for 2017–2018 and Vaccination Coverage Rates for 2015–2016 and 2016–2017 Influenza Seasons.

[8] Martirosyan L., Paget W.J., Jorgensen P., Brown C.S., Meerhoff T.J., Pereyaslov D., Mott J.A. (2012). The Community Impact of the 2009 Influenza Pandemic in the WHO European Region: A Comparison with Historical Seasonal Data from 28 Countries. BMC Infect. Dis..

[9] Statistics Dissemination and Communication Division Statistics Lithuania. https://www.stat.gov.lt/home.

[10] Institute of Hygiene. https://www.hi.lt/structure-and-contacts.html.

[11] Cech D.J., Martin S.T. (2012). Chapter 5—Evaluation of Function, Activity. Functional Movement Development Across the Life Span.

[12] Kuliese M., Jancoriene L., Grimalauskaite R., Zablockiene B., Damuleviciene G., Velyvyte D., Lesauskaite V., Ambrozaitis A., Mickiene A., Gefenaite G. (2017). Seasonal Influenza Vaccine Effectiveness against Laboratory-Confirmed Influenza in 2015–2016: A Hospital-Based Test-Negative Case-Control Study in Lithuania. BMJ Open.

[13] Republic of Lithuania Law Amending Law No VIII-1679 on Ethics of Biomedical Research. https://e-seimas.lrs.lt/portal/legalAct/lt/TAD/76582f93e9c811e59b76f36d7fa634f8?jfwid=181l7lijfh.

[14] World Medical Association Declaration of Helsinki, Ethical Principles for Scientific Requirements and Research Protocols. https://www.wma.net/policies-post/wma-declaration-of-helsinki-ethical-principles-for-medical-research-involving-human-subjects/.

[15] Broberg E., Melidou A., Prosenc K., Bragstad K., Hungnes O. (2016). Region, on behalf of the WHO European Region and the European Influenza Surveillance Network members of the reporting countries. Predominance of Influenza A(H1N1)Pdm09 Virus Genetic Subclade 6B.1 and Influenza B/Victoria Lineage Viruses at the Start of the 2015/16 Influenza Season in Europe. Eur. Surveill.

[16] Melidou A., Hungnes O., Pereyaslov D., Adlhoch C., Segaloff H., Robesyn E., Penttinen P., Olsen S.J. (2020). Predominance of Influenza Virus A(H3N2) 3C.2a1b and A(H1N1)Pdm09 6B.1A5A Genetic Subclades in the WHO European Region, 2018–2019. Vaccine.

[17] Tenforde M.W., Chung J., Smith E.R., Talbot H.K., Trabue C.H., Zimmerman R.K., Silveira F.P., Gaglani M., Murthy K., Monto A.S. (2020). Influenza Vaccine Effectiveness in Inpatient and Outpatient Settings in the United States, 2015–2018. Clin. Infect. Dis. Off. Publ. Infect. Dis. Soc. Am..

[18] Stuurman A.L., Bollaerts K., Alexandridou M., Biccler J., Díez Domingo J., Nohynek H., Rizzo C., Turunen T., Riera-Montes M. (2020). Vaccine Effectiveness against Laboratory-Confirmed Influenza in Europe—Results from the DRIVE Network during Season 2018/19. Vaccine.

[19] Castilla J., Portillo M.E., Casado I., Pozo F., Navascués A., Adelantado M., Gómez Ibáñez C., Ezpeleta C., Martínez-Baz I. (2020). Effectiveness of the Current and Prior Influenza Vaccinations in Northern Spain, 2018–2019. Vaccine.

[20] Puig-Barbera J., Guglieri-Lopez B., Tortajada-Girbes M., Lopez-Labrador F.X., Carballido-Fernandez M., Mollar-Maseres J., Schwarz-Chavarri G., Baselga-Moreno V., Mira-Iglesias A., Diez-Domingo J. (2017). Low Influenza Vaccine Effectiveness and the Effect of Previous Vaccination in Preventing Admission with A(H1N1)Pdm09 or B/Victoria-Lineage in Patients 60 Years Old or Older during the 2015/2016 Influenza Season. Vaccine.

[21] Davies J.R., Grilli E.A. (1989). Natural or Vaccine-Induced Antibody as a Predictor of Immunity in the Face of Natural Challenge with Influenza Viruses. Epidemiol. Infect..

[22] (2017). European Centre for Disease Prevention and Control. Influenza Virus Characterisation, Summary Europe, 2017; Stockholm.

[23] Melidou A., Broberg E. (2017). Predominance of Influenza A(H3N2) Virus Genetic Subclade 3C.2a1 during an Early 2016/17 Influenza Season in Europe—Contribution of Surveillance Data from World Health Organization (WHO) European Region to the WHO Vaccine Composition Consultation for No. Vaccine.

[24] Trebbien R., Fischer T.K., Krause T.G., Nielsen L., Nielsen X.C., Weinreich L.S., Lis-Tønder J., Skov M.N., Christiansen C.B., Emborg H.-D. (2017). Changes in Genetically Drifted H3N2 Influenza A Viruses and Vaccine Effectiveness in Adults 65 Years and Older during the 2016/17 Season in Denmark. J. Clin. Virol..

[25] Castilla J., Martinez-Baz I., Navascues A., Casado I., Aguinaga A., Diaz-Gonzalez J., Delfrade J., Guevara M., Ezpeleta C. (2018). Comparison of Influenza Vaccine Effectiveness in Preventing Outpatient and Inpatient Influenza Cases in Older Adults, Northern Spain, 2010/11 to 2015/16. Eur. Surveill. Bull. Eur. Sur Les Mal. Transm. Eur. Commun. Dis. Bull..

[26] Mira-Iglesias A., López-Labrador F.X., Guglieri-López B., Tortajada-Girbés M., Baselga-Moreno V., Cano L., Mollar-Maseres J., Carballido-Fernández M., Schwarz-Chavarri G., Díez-Domingo J. (2018). Influenza Vaccine Effectiveness in Preventing Hospitalisation of Individuals 60 Years of Age and over with Laboratory-Confirmed Influenza, Valencia Region, Spain, Influenza Season 2016/17. Eur. Surveill. Bull. Eur. Sur Les Mal. Transm. Eur. Commun. Dis. Bull..

[27] Rizzo C., Gesualdo F., Loconsole D., Pandolfi E., Bella A., Orsi A., Guarona G., Panatto D., Icardi G., Napoli C. (2020). Moderate Vaccine Effectiveness against Severe Acute Respiratory Infection Caused by A(H1N1)Pdm09 Influenza Virus and No Effectiveness against A(H3N2) Influenza Virus in the 2018/2019 Season in Italy. Vaccines.

[28] Wu N.C., Zost S.J., Thompson A.J., Oyen D., Nycholat C.M., McBride R., Paulson J.C., Hensley S.E., Wilson I.A. (2017). A Structural Explanation for the Low Effectiveness of the Seasonal Influenza H3N2 Vaccine. PLoS Pathog..

[29] Gherasim A., Martinez-Baz I., Castilla J., Pozo F., Larrauri A. (2017). Effect of Previous and Current Vaccination against Influenza A(H1N1)Pdm09, A(H3N2), and B during the Post-Pandemic Period 2010–2016 in Spain. PLoS ONE.

[30] McLean H.Q., Thompson M.G., Sundaram M.E., Meece J.K., McClure D.L., Friedrich T.C., Belongia E.A. (2014). Impact of Repeated Vaccination on Vaccine Effectiveness against Influenza A(H3N2) and B during 8 Seasons. Clin. Infect. Dis..

[31] Castilla J., Navascués A., Casado I., Pérez-García A., Aguinaga A., Ezpeleta G., Pozo F., Ezpeleta C., Martínez-Baz I. (2018). Interim Effectiveness of Trivalent Influenza Vaccine in a Season Dominated by Lineage Mismatched Influenza B, Northern Spain, 2017/18. Eur. Surveill. Bull. Eur. Sur Les Mal. Transm. Eur. Commun. Dis. Bull..

[32] Chan Y.D., Wong M., Au K., Chuang S. (2019). Seasonal Influenza Vaccine Effectiveness at Primary Care Level, Hong Kong SAR, 2017/2018 Winter. Hum. Vaccin. Immunother..

[33] Rolfes M.A., Flannery B., Chung J.R., O’Halloran A., Garg S., Belongia E.A., Gaglani M., Zimmerman R.K., Jackson M.L., Monto A.S. (2019). Effects of Influenza Vaccination in the United States During the 2017-2018 Influenza Season. Clin. Infect. Dis. Off. Publ. Infect. Dis. Soc. Am..

[34] Pebody R., Djennad A., Ellis J., Andrews N., Marques D.F.P., Cottrell S., Reynolds A.J., Gunson R., Galiano M., Hoschler K. (2019). End of Season Influenza Vaccine Effectiveness in Adults and Children in the United Kingdom in 2017/18. Eur. Surveill. Bull. Eur. Sur Les Mal. Transm. Eur. Commun. Dis. Bull..

[35] European Centre for Disease Prevention and Control Influenza in Europe, Summary of the Season 2017–2018. https://www.ecdc.europa.eu/en/seasonal-influenza/season-2017-18.

[36] Adlhoch C., Snacken R., Melidou A., Ionescu S., Penttinen P. (2018). Dominant Influenza A(H3N2) and B/Yamagata Virus Circulation in EU/EEA, 2016/17 and 2017/18 Seasons, Respectively. Eur. Surveill. Bull. Eur. Sur Les Mal. Transm. Eur. Commun. Dis. Bull..

[37] Skowronski D.M., Chambers C., De Serres G., Sabaiduc S., Winter A.-L., Dickinson J.A., Gubbay J.B., Drews S.J., Fonseca K., Charest H. (2019). Vaccine Effectiveness Against Lineage-Matched and -Mismatched Influenza B Viruses Across 8 Seasons in Canada, 2010-2011 to 2017–2018. Clin. Infect. Dis. Off. Publ. Infect. Dis. Soc. Am..

[38] McLean H.Q., Thompson M.G., Sundaram M.E., Kieke B.A., Gaglani M., Murthy K., Piedra P.A., Zimmerman R.K., Nowalk M.P., Raviotta J.M. (2015). Influenza Vaccine Effectiveness in the United States during 2012–2013: Variable Protection by Age and Virus Type. J. Infect. Dis..

[39] Tricco A.C., Chit A., Soobiah C., Hallett D., Meier G., Chen M.H., Tashkandi M., Bauch C.T., Loeb M. (2013). Comparing Influenza Vaccine Efficacy against Mismatched and Matched Strains: A Systematic Review and Meta-Analysis. BMC Med..

[40] Esposito S., Bonanni P., Maggi S., Tan L., Ansaldi F., Lopalco P.L., Dagan R., Michel J.-P., van Damme P., Gaillat J. (2016). Recommended Immunization Schedules for Adults: Clinical Practice Guidelines by the Escmid Vaccine Study Group (EVASG), European Geriatric Medicine Society (EUGMS) and the World Association for Infectious Diseases and Immunological Disorders (WAidid). Hum. Vaccin. Immunother..

[41] Reber A.J., Chirkova T., Kim J.H., Cao W., Biber R., Shay D.K., Sambhara S. (2012). Immunosenescence and Challenges of Vaccination against Influenza in the Aging Population. Aging Dis..

[42] DiazGranados C.A., Dunning A.J., Kimmel M., Kirby D., Treanor J., Collins A., Pollak R., Christoff J., Earl J., Landolfi V. (2014). Efficacy of High-Dose versus Standard-Dose Influenza Vaccine in Older Adults. N. Engl. J. Med..

[43] Darvishian M., van den Heuvel E.R., Bissielo A., Castilla J., Cohen C., Englund H., Gefenaite G., Huang W.-T., la Bastide-van Gemert S., Martinez-Baz I. (2017). Effectiveness of Seasonal Influenza Vaccination in Community-Dwelling Elderly People: An Individual Participant Data Meta-Analysis of Test-Negative Design Case-Control Studies. Lancet. Respir. Med..

[44] Russell K., Chung J.R., Monto A.S., Martin E.T., Belongia E.A., McLean H.Q., Gaglani M., Murthy K., Zimmerman R.K., Nowalk M.P. (2018). Influenza Vaccine Effectiveness in Older Adults Compared with Younger Adults over Five Seasons. Vaccine.

[45] Suess T., Remschmidt C., Schink S.B., Schweiger B., Heider A., Milde J., Nitsche A., Schroeder K., Doellinger J., Braun C. (2012). Comparison of Shedding Characteristics of Seasonal Influenza Virus (Sub)Types and Influenza A(H1N1)Pdm09; Germany, 2007–2011. PLoS ONE.

[46] Khoury J., Szwarcwort M., Kra-Oz Z., Saffuri M., Seh K., Yahalomi T., Braun E., Azzam Z.S., Paul M., Neuberger A. (2018). Duration of Viral Shedding and Factors Associated with Prolonged Shedding among Inpatients with Influenza Treated with Oseltamivir: A Prospective Cohort Study. Eur. J. Clin. Microbiol. Infect. Dis. Off. Publ. Eur. Soc. Clin. Microbiol..

[47] De Serres G., Skowronski D.M., Wu X.W., Ambrose C.S. (2013). The Test-Negative Design: Validity, Accuracy and Precision of Vaccine Efficacy Estimates Compared to the Gold Standard of Randomised Placebo-Controlled Clinical Trials. Eur. Surveill..

[48] Sullivan S.G., Feng S., Cowling B.J. (2014). Potential of the Test-Negative Design for Measuring Influenza Vaccine Effectiveness: A Systematic Review. Expert Rev. Vaccines.

[49] Foppa I.M., Haber M., Ferdinands J.M., Shay D.K. (2013). The Case Test-Negative Design for Studies of the Effectiveness of Influenza Vaccine. Vaccine.

